# The Need for a Bleed Type–Specific Annual Bleeding Rate in Hemophilia Studies

**DOI:** 10.2196/51372

**Published:** 2024-03-13

**Authors:** Kun Huang

**Affiliations:** 1 Department of Pediatrics The First Affiliated Hospital Zhejiang University School of Medicine Hangzhou China

**Keywords:** benefit-risk assessment, discrete choice experiment, hemophilia A, patient preference, prophylactic treatment

We read with great interest the paper “Quantifying benefit-risk trade-offs toward prophylactic treatment among adult patients with hemophilia A in China: discrete choice experiment study” by Wang et al [1]. The results were unsurprising and aligned with clinical practice and provided valuable insights into the preferences of patients for prophylaxis in hemophilia A treatment. The authors designated the annual bleeding rate as an important item in the questionnaire used. This is rational as most clinical studies on hemophilia consider it a primary clinical outcome. However, there is a need for greater specificity in the annual bleeding rate, particularly with regard to joint bleeding and spontaneous bleeding.

As the standard treatment for patients with hemophilia A, prophylaxis with coagulation factor VIII (FVIII) was proposed to maintain a steady trough FVIII level mainly to prevent spontaneous and breakthrough bleeds [2]. In the study, the annual bleeding rate was calculated using all types of bleeds, including injury-caused, spontaneous, joint, and subcutaneous bleeds. However, a great difference exists across these bleeding types. For example, compared with subcutaneous bleeds, joint bleeds typically result in more serious consequences and require greater attention and more effective management. The definition of “target joint” only needs 3 spontaneous bleeds to occur in a single joint [2]. However, patients with target joints will need a substantially higher FVIII level to ensure their safety [3]. On the other hand, it is common for patients with active lifestyles to report more mild injury-caused bleeds (eg, bleeding resulting from a sports-related injury) but a good quality of life and normal joint structure or function [4]. As reported before, the primary aim of prophylaxis is to prevent spontaneous bleeds, which could be completely different from injury-caused bleeds [2]. All individuals can bleed if an injury occurs while taking part in sports; thus, sports-related bleeds are not equivalent to spontaneous bleeds [5]. For example, there are substantial differences in treatment or clinical outcome between patients with 6 instances of spontaneous joint bleeds and those with 6 instances of injury-caused bleeds in 1 year. Thus, Wang et al [1] could consider distinguishing bleed types, at least for spontaneous, injury-caused, joint, and other mild bleeds. With reference to the attributes of efficacy factor among the authors’ citations, 3 studies (references 22, 23, and 25) picked reduction of bleeds and 2 studies (references 25 and 26) selected breakthrough bleeds instead of annual bleeding rate. In 2 studies that did not involve specific bleed types, one (reference 28) added joint evaluation to make the results clearer.

In conclusion, although the annual bleeding rate is a commonly used indicator for clinical outcomes among patients with hemophilia, it is better to distinguish different kinds of bleeds or simply choose a more detailed and suitable one for analysis in a discrete choice experiment. This will eliminate potential confusion during the interpretation of results and make study findings and conclusions more detailed and valid, thus enhancing their clinical application.

## Abbreviations

FVIII: factor VIII
